# Plastic and Anthropogenic Particles in the Reproductive Tissues of Feral and Domestic Felines

**DOI:** 10.3390/ani16172804

**Published:** 2026-09-07

**Authors:** Emma Quade, Madison Serrate, Vageeswari Nathapettai Thanikaivelan, Fnu Joshua, Lei Zhai, Frank Logiudice, Linda J. Walters

**Affiliations:** 1 Department of Biology, University of Central Florida, 4000 Central Florida Blvd., Orlando, FL 32816, USA; madison.serrate@ucf.edu (M.S.); frank.logiudice@ucf.edu (F.L.); linda.walters@ucf.edu (L.J.W.); 2 Department of Chemistry, University of Central Florida, 4000 Central Florida Blvd., Orlando, FL 32816, USA; vageeswari.nathapettaithanikaivelan@ucf.edu (V.N.T.); fnu.joshua@ucf.edu (F.J.); lzhai@ucf.edu (L.Z.); 3 Nanoscience and Technology Center, University of Central Florida, 12424 Research Parkway, Orlando, FL 32826, USA; 4 Center for Integrated Coastal Research, University of Central Florida, 4000 Central Florida Blvd., Orlando, FL 32816, USA

**Keywords:** anthropogenic particle, companion animal, cat, plastic pollution, FTIR, PET

## Abstract

Feral and domestic felines are continuously exposed to meso- (polymer length: 5–25 mm) and microplastics (polymer length < 5 mm) from environmental or household contaminants. Ingestion and inhalation are the primary pathways for internalization of plastic, non-plastic anthropogenic (e.g., rayon, AP), and natural cellulose particles. However, contamination information is limited for companion animals. This study focused on reproductive tissue of felines by obtaining samples from spay (ovary + uterus) and neuter (testes) procedures. The tissue was chemically digested and then analyzed via microscopy and Fourier-Transform Infrared Spectroscopy (FTIR) to detect and identify contamination. Overall, 71% of 100 individuals contained particles (meso- and microplastics, non-plastic anthropogenic particles, cellulose), of which 97.3% were fibers. Foreign particles were present in the uterus and ovaries of 100% of domestic females, while only 36% of feral males had particles in their testes. Overall, 25% of the FTIR-confirmed particles were plastic, 54% were AP, and 21% were cellulose. The most common plastic found was polyethylene terephthalate (PET), a common polymer used in packaging food and beverages, including manufactured cat food and bottled water. This study provides a baseline for understanding plastic and AP concentrations in the reproductive tissues of both feral and domestic felines.

## Simple Summary

Although plastics are useful in modern society, once they enter the environment, they can be inhaled or ingested by terrestrial and aquatic vertebrates and invertebrates. While microplastics (MP) (length: <5 mm), mesoplastics (MSP) (length: 5–25 mm), non-plastic anthropogenic particles (AP) (e.g., rayon), and cellulose have been identified in tissues of organisms, information is limited for companion animals, especially in their reproductive tissues. Our study assessed plastic/AP/cellulose contamination in the reproductive tissues of feral and domestic felines. In total, 50 males and 50 females were examined, and we predicted that feral females would have a greater presence of particles due to increased blood flow through reproductive tissue and the known outdoor levels of MP. Tissue samples were collected, chemically processed, and examined via microscopy and spectroscopy. A total of 71% of felines had foreign particles present in their reproductive tissues. Of all particles tested and identified, 25% were plastic, 54% were AP, and 21% were cellulose. Domestic females had the greatest presence of particles (100%) in their tissues and feral males had the fewest (36%), with polyethylene terephthalate confirmed in all tested feline categories. Tissue-specific differences and lifestyles provide suggestions on ways to limit future contamination in feline companion animals.

## 1. Introduction

Due to the durability, flexibility, and cost-effectiveness of plastic materials, they have become essential in today’s world [1]. Plastics, however, when exposed to the environment, can break down into microplastics (MP), particles less than 5 mm in size, which can cause adverse effects on both the environment and contaminated organisms [2]. Plastic particles larger than MP (5 mm–25 mm) are known as mesoplastics (MSP), and plastics 25 mm–1 m are macroplastics [3]. Particle morphologies include fragments, fibers, films, beads, and foams, with fibers being the most abundant form [4]. MP are also categorized as primary or secondary. Particles manufactured originally at this small size are considered primary MP or MSP, while secondary MP/MSP have fragmented from larger plastic pieces [5]. MP and MSP are easily inhaled or ingested by organisms, leading to these contaminants infiltrating their tissues [6]. Other synthetic or semi-synthetic particles, known as anthropogenic particles (AP), are primarily shed from manufactured textiles. AP also pollute ecosystems and can be inhaled or ingested by organisms in a similar manner as MP [7].

Plastic particles have been discovered in the digestive and respiratory systems of numerous organisms, with ingestion and inhalation suggested as primary entryways. MP have also been found in other organs (e.g., kidney, liver), demonstrating their ability to translocate through tissue barriers in vertebrates [8]. For example, studies have detected MP in male and female human reproductive tissues [9,10]. In the human female uterus, MP caused significant adverse effects such as toxicity in endometrial organoids and induced cell apoptosis [8]. In human males, MP disrupt the blood–testis barrier, lead to decreased sperm quality, and impact early embryo development [10]. In felines, MP were detected in placental and fetal tissue, suggesting the ability of MP to cross the placental barrier, predisposing young felines to be born with MP in their tissues [11]. Another study focused on companion animals detected MP in lungs, ileum, liver, kidney, and in blood clots of both canines, *Canis familiaris*, and felines, *Felis catus* [8].

While feral and domestic felines of *F. catus* are physically and biologically identical, their lifestyles are usually distinct, resulting in individuals being exposed to different contaminants. Comparing plastic densities in their tissues could lead to potential source theories of the origin of these particles, potentially aiding in lowering future exposure rates. The diets of feral and domestic felines typically differ, with most domestic felines given manufactured cat food. MP contamination has been reported in manufactured feline food [12], as well as in the lining of cat food cans [13], suggesting the potential for domestic felines to consume MP in their diets. Feral felines prey in the wild on small mammals, birds, reptiles, amphibians, invertebrates, and fish [14]. MP contamination has been documented in all of these prey types [15,16,17,18,19], making it plausible that felines consume MP by consuming live prey.

Water sources and aerial contamination may also contribute to MP contamination. Freshwater environments including lakes, rivers, and collected rainwater have been found to have MP contamination [20,21]; these are common sources from which feral felines drink. Alternatively, MP have also been detected in domestic feline water sources including bottled and tap water [22]. When considering contamination via inhalation, both feral and domestic felines may inhale MP. Recent studies have found there to be a significantly greater amount of MP in the air of indoor spaces compared to outdoors, which may be attributed to furniture, carpeting, and textiles [23].

Since the uterus and ovaries in female felines and the testes in male felines play essential roles in nourishing the embryo and reproduction [24], it is important to examine the presence of MP and other foreign particles within these organs. Studying how plastic particles are absorbed, transported, and behave in the uterus and ovaries as well as in the testicles would be crucial for understanding their potential effects on fertility and overall reproductive function. The primary objective of this study was to provide baseline information on plastic contamination in reproductive tissues of felines. Additionally, we examined all types of particles encountered, including MP, MSP, AP, and cellulose. We identified and compared particle presence in the reproductive tissues in feral versus domestic felines, compared particle presence in the uterus and ovaries with presence in testes, and determined if the collected tissue weight as well as the age and weight of felines influenced particle presence in the reproductive tissues of domestic felines.

## 2. Materials and Methods

### 2.1. Sample Collection

For this study, reproductive tissues from feral and domestic felines (*Felis catus*) were obtained from Pet Alliance of Greater Orlando, a non-profit spay/neuter clinic that partners with Orange County, FL, USA Animal Services. Domestic felines were brought in by clients living in Orlando, Ocoee, Longwood, Winter Garden, Apopka, Winter Park, and Oviedo, all residing within a 30-mile radius of the clinic. Feral felines were brought in from various, mostly unknown, locations throughout Orange County. In total, 100 tissue samples were collected; this included 25 from domestic males and 25 from domestic females, as well as 25 from feral males and 25 from feral females. Tissue samples were obtained following ovariohysterectomies of the females, during which both ovaries and the uterus were removed and collected simultaneously, and following castrations of the males, during which both testes were removed and collected. All surgeries were performed by licensed veterinarians. Prior to starting the project, our university IACUC office informed us that their approval was not required for this research because: (1) the procedures were conducted off campus by non-university staff, (2) the procedures were not conducted specifically for our research, and (3) only discarded material destined for the trash was used in our study [25].

Aerial contamination in the surgical room was monitored during the procedures using procedural contamination blanks. Five blanks (open Petri dishes that had been triple-rinsed with 0.45 µm filtered deionized water and contained a 0.45 µm filter paper dampened with 0.45 µm filtered deionized water) were set up around the perimeter of the surgery room, approximately one meter from each surgical table and approximately 1.5 m from the nearest other Petri dish. For consistency, procedural blanks were placed in the same locations on every collection day and were replaced with new blanks every hour for the duration of the procedures. The samples were collected using non-plastic, sterile surgical instruments, weighed, placed in 40 mL glass vials, and then frozen at −40 °C until processing. Each vial, as well as the rest of the glassware, Petri dishes, and equipment utilized, was triple-rinsed with deionized (DI) water previously filtered through 0.45 µm filters, following established microplastic research guidelines prior to sample collection [26]. All collected samples were tissues that were previously removed from the feline’s body and would have otherwise been discarded. Information about the felines, including their sex, age (for domestics), and body weight, was documented (Table 1).

### 2.2. Tissue Digestion

To chemically digest the samples, the tissue mass was recorded using a digital balance (Ohaus SP-6001 Scout Pro, Parsippany, NJ, USA) before transferring the tissue into individual 125 mL glass Erlenmeyer flasks that had been triple-rinsed with 0.45 µm filtered DI water. For females, the uterus and ovaries from each feline were pooled, and the total mass was recorded, with both organs placed in one flask. For males, the total mass of both testes was recorded and pooled in the same flask. A prepared 10% potassium hydroxide (KOH) solution (Thermo Fisher Scientific, Pittsburgh, PA, USA) made with 0.45 µm filtered DI water was added to each flask at a ratio of 3:1, three times the biological mass of the tissue sample [27]. KOH has been found to be the preferred chemical used for chemical digestion of organic materials as it was not destructive to most plastics [28].

Samples were then placed in a Labnet 311DS Environmental Shaking Incubator (115V Model # I-5311-DS, Edison, NJ, USA) at 65 °C and 65 rpm for 48 h. After 48 h, samples were removed from the incubator and left to sit at room temperature (21 °C) for an additional 24 h [29]. A 10 M citric acid solution (Thermo Fisher Scientific, Pittsburgh, PA, USA) was then added to each sample to neutralize the KOH to a pH of 7, using pH test strips to measure pH. This was done to prevent degradation of the filter papers used during the filtration process [30]. Each sample was then filtered using a GAST vacuum pump (Gast Manufacturing, Inc., Benton Harbor, MI, USA) through gridded (9 mm^2^), 0.45 µm nitrocellulose membrane filter papers [31]. These filter papers allowed the dissolved feline tissue to pass through and caught any potential contaminants to then be analyzed.

### 2.3. Microscopy Analysis

**Figure 1:**
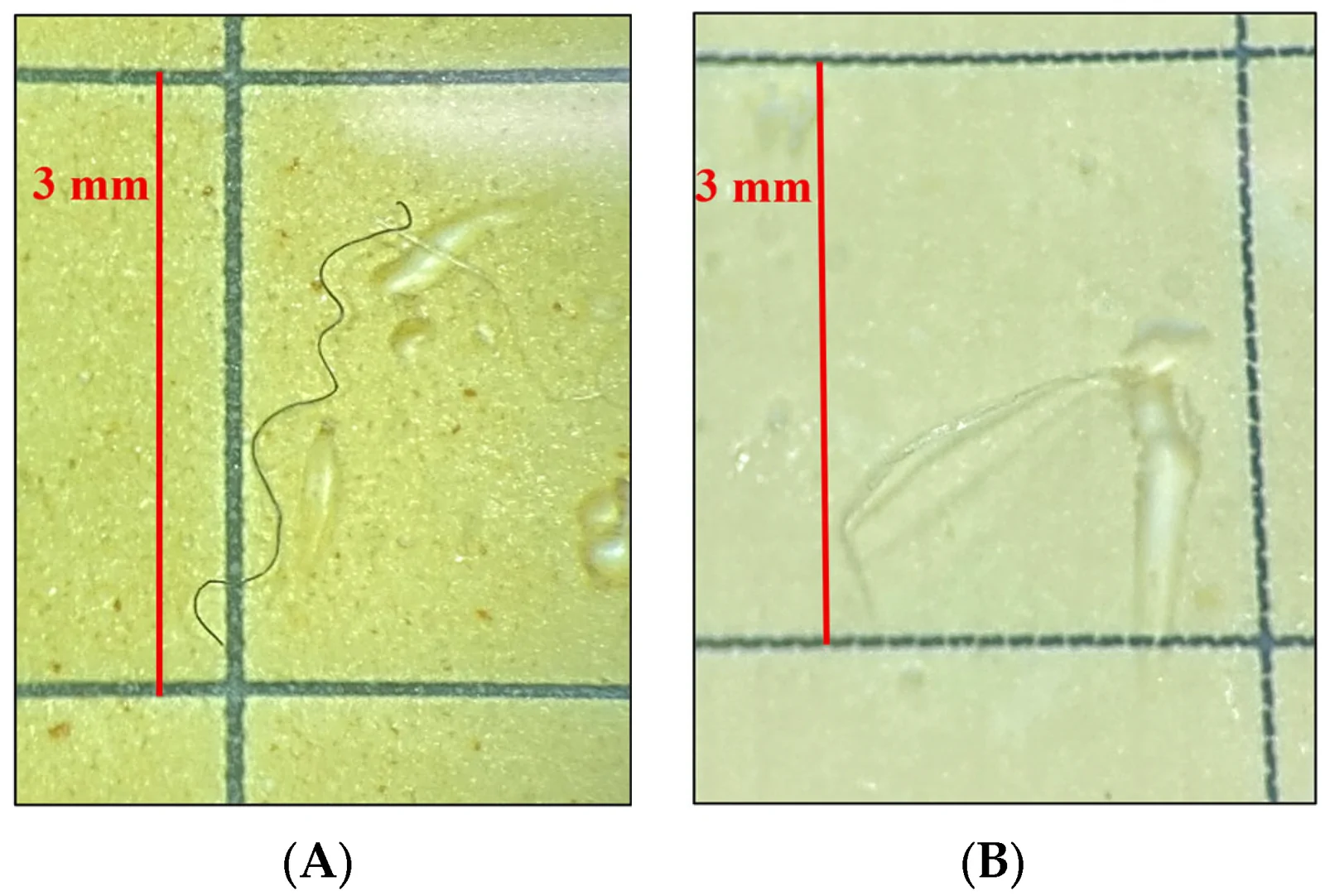
Microscopy images. (**A**) Black Microplastic Fiber (PET) from Feral Female Feline; (**B**) Clear Microplastic Fiber (PET) from Domestic Male Feline. (Red line is 3 mm as the reference).

The 0.45 µm filter papers used for vacuum filtration were placed in individual triple-rinsed Petri dishes and analyzed for MP and other particles under a Leica EZ4 dissecting microscope (Leica Microsystems, Wetzlar, Germany) at 20–40× magnification [32]. For any particles that were found, information about their size, color, and shape was recorded (Figure 1).

### 2.4. Limiting Contamination in the Laboratory

Five procedural contamination blanks (0.45 µm filtered DI water triple-rinsed Petri dishes containing a 0.45 µm filter paper dampened with 0.45 µm filtered DI water) surrounded the microscope (~15 cm from the microscope), were open while the samples were exposed, and analyzed via microscopy to account for any aerial contamination during microscopy [33]. These procedural blanks were analyzed to record the aerial particle contamination per minute. The contamination rates were calculated by finding the average exposure time per sample and dividing that by the average number of particles found in each blank. No procedural blanks were used during the digestion procedures as the flasks were continuously covered with aluminum foil at all times to avoid aerial contamination. The neutralization step was completed in a fume hood in less than 20 s, and samples were immediately recovered with aluminum foil. During filtration, samples were kept covered with aluminum foil and lids were utilized on the filtration apparatuses. In addition, 100% cotton clothing was worn at all times during the research process.

### 2.5. Fourier-Transform Infrared Spectroscopy

Fourier-transform infrared spectroscopy (FTIR) analysis was used to identify the molecular composition of the identified particles [31]. This technique was conducted at the University of Central Florida Nanoscience Technology Center and was performed using an Alpha II compact FT-IR spectrometer with attenuated total reflection (ATR) accessories (Bruker, Ettlingen, Germany) and the KnowItAll IR Spectral Library Collection for matching analysis. A random number generator was utilized to select a subset containing 41% (n = 91) of the 220 total particles found, while ensuring representation of all groups. While replicate FTIR measurements were not performed on individual particles [31], the use of 40 averaged scans together with spectral matching provided confidence in the identification of each environmental MP sample. We recognize the absence of independent replicate measurements can be considered a limitation to our study. Each spectrum was smoothed prior to library matching, and only results that had a 70% or higher match score with the database were deemed reliable [33].

### 2.6. Statistical Analysis

All statistical analyses were performed using RStudio software version R 4.4.3 [34]. Analysis was conducted on particles found via microscopy. The “dredge” function from the MuMIn package (version 1.48.19) was used to perform AIC-based model selection on models with multiple predictors [35]. Contaminating particle presence/absence for feline reproductive tissue across groups was analyzed using a binomial generalized linear model. Feline weight and collected tissue weight were included in the final model (GLM Binomial: Particle Presence ~ Status + Reproductive Tissue Type + Cat Weight + Tissue Weight). Pairwise comparisons of particle contamination presence/absence between groups were conducted using Tukey’s post hoc method from the emmeans package (version 1.11.2-80003) [36]. To avoid speculation, age was excluded from the above models as feral cat age was estimated by the veterinarian. To assess age and particle presence in domestic felines, a Firth-penalized logistic regression (logistf package, version 1.26.1) was fit to account for high particle presence in domestic cats (Firth Binomial: Particle Presence ~ Age Centered * Reproductive Tissue Type) [37]. Age analysis was performed on domestic females and males across only with age centered. Goodness of fit for all GLMs was examined using the Hosmer–Lemeshow test. All models were assessed visually using diagnostic plots and indicated no major violations.

## 3. Results

### 3.1. Particle Contamination: Feral vs. Domestic, Male vs. Female

**Figure 2:**
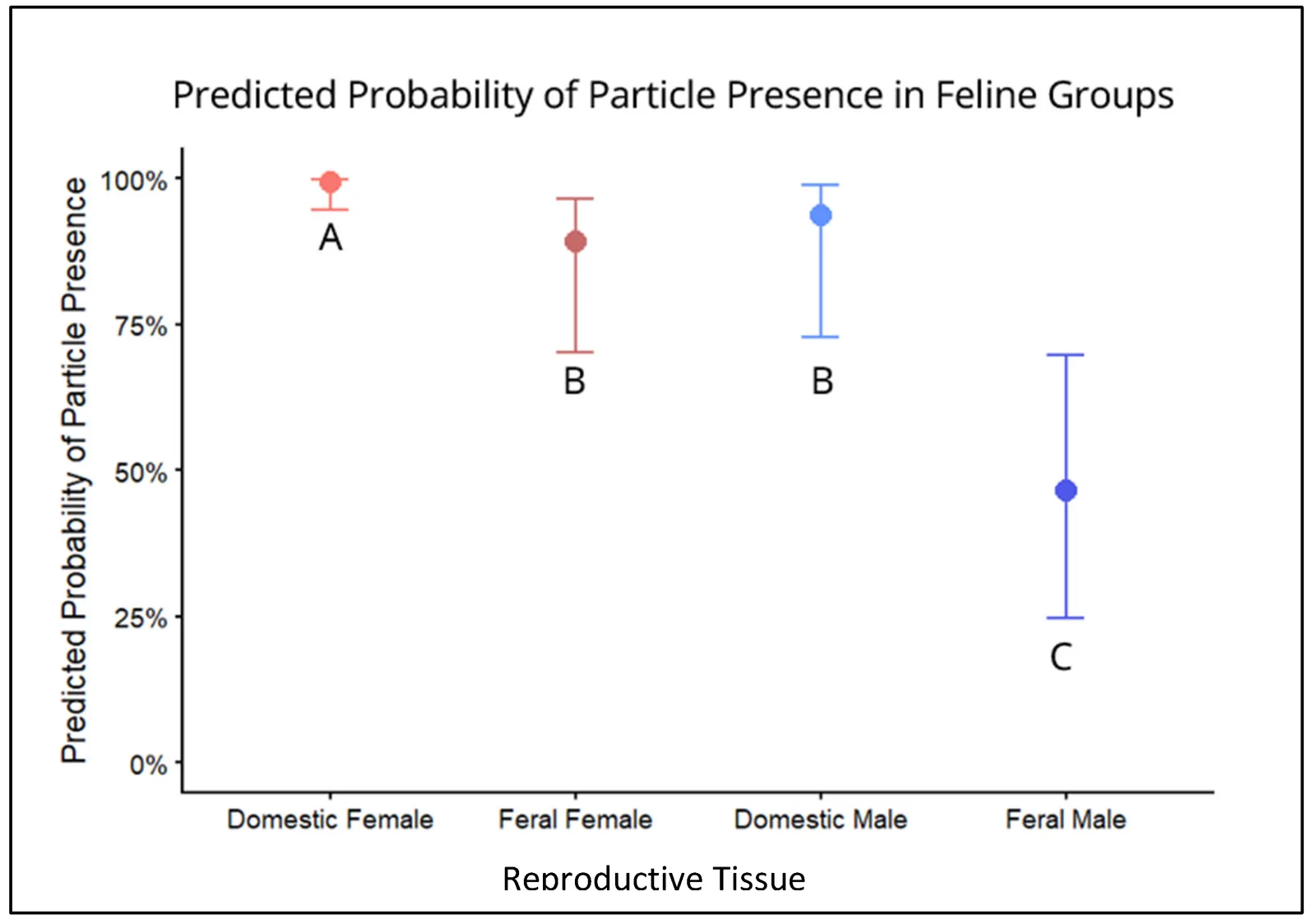
Predicted probability of particle presence from GLM Binomial: Particle Presence ~ Status + Reproductive Tissue + Cat Weight + Tissue Weight. Pairwise Tukey Test: Letters denote significant differences between groups (A > B > C). Female = pooled uterus plus ovaries. Male = pooled testes.

A total of 220 particles were recorded as contaminating particles in the reproductive tissues of 100 felines. In total, 71% of felines tested contained particles in their reproductive tissues. This included 100% of domestic feline uteri plus ovaries, 80% of feral feline uteri and ovaries, 68% of domestic feline testes, and 36% of feral feline testes (Figure 2). Of these particles, 97.3% were fibers, ~2% were fragments, and less than 1% were films (Table 1).

Feral felines were found to have significantly fewer individuals with particles than domestic felines (Binomial GLM; *p* < 0.001; β = −2.82). Testes had less particle presence overall than pooled uteri plus ovaries (Binomial GLM; *p* = 0.002; β = −2.24). Greater reproductive tissue weight was positively correlated with particle presence (Binomial GLM; *p* = 0.004; β = 1.49), with the odds of particle presence increasing as tissue weight increased. Feline body weight, however, was negatively correlated with particle presence (Binomial GLM; *p* = 0.018; β = −0.758). A Hosmer–Lemeshow test was non-significant (*p* = 0.423), indicating our model fit the data. An interaction between status and reproductive tissue type was initially included in the full presence/absence model but was non-significant (*p* = 0.992). This interaction did not improve model performance and was subsequently dropped.

### 3.2. Plastics and Anthropogenic Particles: Accumulation with Age

**Figure 3:**
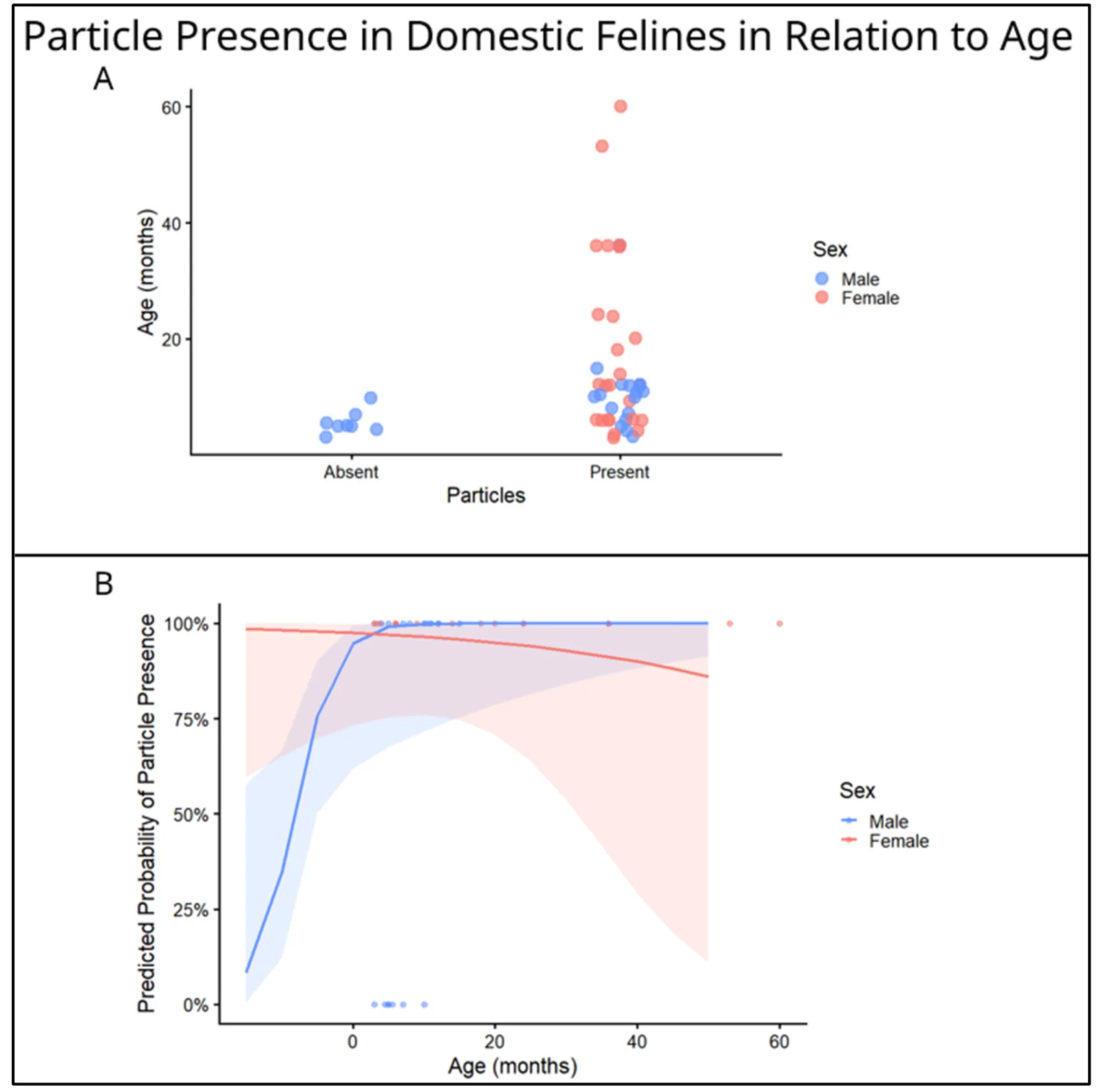
(**A**) Raw data showing presence/absence of particles in domestic males and females across ages. (**B**) Predicted Particle Presence versus Age in Domestic Felines: Particle Presence ~ Age Centered * Reproductive Tissue Type. Dots represent raw data. Note the age scale is centered at average age rather than zero.

Firth logistic regression results show that there was a significant interaction between age and reproductive tissue type for particle presence (Firth Binomial: *p* = 0.039; β = −0.389). For males, the probability of particle presence increased significantly with age (Firth Binomial: *p* = 0.018; β = 0.352), with particles being present in 17/25 domestic feline testes (Figure 3). Particles were present in all domestic feline uteri plus ovaries (25/25); this limited the ability to detect any relationship between age and particle presence (β = −0.037). We acknowledge the limited interpretability of the age-related models given the small dataset (domestic felines only).

### 3.3. FTIR Results

FTIR was performed on a randomly selected subset consisting of 41% (n = 91) of the 220 particles. Out of this 41%, 5% (n = 11) of the particles were deemed unusable during the FTIR process due to lack of library validation, resulting in an FTIR spectrum at 70% or greater obtained for 36% (n = 80) of the particles. FTIR spectra confirmed that 25% (n = 20) of these 80 particles were plastic polymers (MP: 10, MSP: 10) (Table 1). Of the remaining particles, 54% (n = 43) were AP (Table 2) and 21% (n = 17) were natural plant cellulose.

Of the 20 FTIR-validated plastic particles, 100% of them were fibers within the range of 1–12 mm, having an average length of 4.75 ± 0.79 mm (SE). The color distributions of the validated MP/MSP were 55% clear, 15% black, 15% navy blue, 10% pink, and 5% light blue.

The FTIR match material results (Table 2) revealed that polyethylene terephthalate (PET) was the most prevalent polymer found in this study (n = 9 particles) and was found in at least one feline from each group—domestic male and female, feral male and female. Polyurethane (PU) (n = 5) was found in domestic males, feral females, and feral males. Polyethylene fibers (n = 2) were in a feral female and a feral male, polyester (n = 2) was observed in a domestic male and feral female, and poly (acrylic acid/starch) grafted resin fibers (n = 2) were found in two separate feral female reproductive tissues. Ten of these FTIR-validated plastics were MP and 10 were MSP. PET was the most common plastic polymer observed for both MP (n = 5) and MSP (n = 4), and PU was next most common for both particle types: MP (n = 3) and MSP (n = 2). Polyester was only observed as MSP (n = 2), and there was 1 MP fiber and 1 MSP fiber of each Polyethylene and poly (acrylic acid/starch) grafted resin.

Of the AP found, carboxymethyl cellulose (n = 20) was the most prevalent and was observed in domestic males and females and feral females (Table 2). Hydroxyethyl cellulose was seen in a lower quantity (n = 15); however, it was seen in all four feline groups. Thickener L (polymethylvinylether/maleic anhydride) (n = 5) was also found throughout all four feline groups, while Rayon-Absorbent (n = 3) was only found in feral females.

There were 7 different cellulose library matches detected. These variants are all cellulosic material and are all classified as natural fibers in the FTIR library.

### 3.4. Contamination Rate Results

Aerial contamination during the surgical procedures was calculated to be 0.0003 ± 0.017 particles/min, with each blank having an exposure time of 60 min. In total, there was only one particle (length: 1 mm) observed in the blanks placed in the surgical room, which was clear in color. The contamination rate during microscopy was calculated at 0.0005 ± 0.003 particles/min, with an average exposure time of 11.7 ± 0.4 min. There were 3 particles observed in the microscopy blanks that had lengths of 2 mm, 2 mm, and 3 mm. Particle colors were clear, navy, and black, respectively. Following the literature [38], both low values were considered negligible, and therefore no contamination correction factor was included in the data analysis. FTIR was not conducted on these 4 particles, and we understand that this is a limitation of our study.

## 4. Discussion

When considering the effect of MP and other polluting particles on companion animals, such as cats and dogs, there is little research on the abundance of these pollutants in their internal systems. Given that over 70% of people in the U.S. own at least one companion animal [39], it is important to acknowledge how they may be affected. In other mammals, the reproductive system has been found to be sensitive to MP and other environmental pollutants, even in trace amounts and at fetal life stages [40]. This contamination has led to complications including endocrine disruption, epigenetic changes, oxidative stress, and impairment of innate immune defenses [40]. With the recent discovery of the ability of MP to cross the placental barrier and invade fetuses in felines [11], concerns have increased regarding the overall reproductive health of *F. catus*.

The plastic polymers found in this study are widely used today. PET, a type of polyester and the most prevalent plastic found in this study, is the most widely used plastic for packaging food and beverages, and there are concerns about the health effects of consumers in contact with this material [41]. PET has also been discovered in both manufactured dry and wet cat foods [12]. In this study, PET was confirmed in all four feline groups. PU is a popular polymer due to its cost-effectiveness and energy-saving properties, and is commonly found in upholstered furniture, insulation in walls and roofs, medical devices, and automotive interiors [42]. Another plastic material detected was polyethylene, which can be found in packaging films, rigid containers, and pipes [43]. Poly (acrylic acid/starch) grafted resin is known as a superabsorbent polymer designed for liquid absorption, found in various hygiene products that have now become prevalent in landfills, drinking water, and human bloodstreams [44]. While our limited sample size prevented recognition of any clear associations between groups of felines and different polymers present in their reproductive tissue, feral ovaries and uteri had the greatest diversity of polymers present, containing all five types identified in this study.

The AP detected in this study could also be attributed to a variety of sources. Many of these substances, including carboxymethyl cellulose, hydroxyethyl cellulose (both cellulose derivatives), and thickener L (polymethylvinylether/maleic anhydride), are used as thickeners for a wide variety of substances in food production, soaps, and detergents, as well as for industrial applications [45,46,47]. Rayon-absorbent is a material found in fabrics used in clothing and other materials [48]. It is also possible that the cellulose detected from the environment may have undergone oxidation by weathering, causing the detected cellulose to be labeled as other cellulose derivatives instead of pure cellulose. With these man-made materials being prevalent in the environment and the internal systems of animals, future studies should continue to investigate possible health implications of these particles.

A significantly greater presence of MP were found in the reproductive tissues of domestic felines, which was contrary to our original hypothesis. Possible explanations could be due to the different lifestyles of feral and domestic felines, including their food and water sources, as well as their environment. In addition to PET discovered directly in manufactured cat food [12], one study found that 79% of cat owners use plastic containers to store their cat food [49], potentially increasing MP contamination from shedding. One of the most common polymers used for food storage containers is PET [50], which was the most prevalent polymer found throughout this study. Using containers of other materials could eliminate plastic shedding during storage [51], and potentially lower overall plastic consumption.

Another possible contributor to plastic contamination in felines is aerial contamination. Indoor spaces have significantly greater aerial plastic concentrations, with one study finding aerial MP contamination of an indoor apartment and office space to be 1- 60 fibers/m^3^ per day, compared to an urban outdoor space, which had 0.3–1.5 fibers/m^3^ [52]. This significant difference in air quality could result in many of these contaminants entering felines’ internal systems via inhalation. The results of this study indicated polypropylene being the predominant indoor polymer [52]. The plastic materials in the air indoors likely depend on the types of carpeting and textiles used in each individual indoor space. The ability of plastic to disperse once released into the atmosphere lowers the overall concentration in outdoor areas [52], resulting in feral felines having a lesser inhalation potential of contaminants. To combat aerial contamination in indoor spaces, new technology is being developed to filter air and reduce the amount of plastics being inhaled. One filtration product, recently tested, had an 86% collection efficiency of harvesting MP fibers from the atmosphere [53]. Although further research is needed, products such as air filters and purifiers could also be useful in lowering MP aerial contamination.

Plastic cat toys could hypothetically be another contributor to plastic in the reproductive tissues in felines. Many pet toys contain products made of synthetic textiles, such as polyester, which readily shed MP [13]. Pet toys are one of the categories of pet products that the U.S. Food and Drug Administration does not regulate in the USA [54]. Thus, there are no regulations for what materials can or cannot be used to make pet toys, resulting in potentially harmful toys being distributed. Müller et al. [55] reported that veterinarians recognized the threat posed by pets swallowing pieces of toys made of synthetic material, such as polyvinyl chloride. Moreover, even though toys may be initially produced as ‘soft plastics’, after ingestion these pieces often become hard and sharp in the gastrointestinal tract, indicating that any softeners present in the toy leached out of the plastic and into the pet’s internal system [55]. This emphasizes the importance of avoiding pet toys made of synthetic materials, as they not only shed MP, but could also pose physical threats to the feline’s gastrointestinal tract.

A structure that could potentially explain lower MP presence in testes is the blood–testis barrier. This barrier around the testes contains tight junctions known as Sertoli cell junctions [56]. This resistant biological barrier is known to be one of the ‘tightest’ in the mammalian body [56] and serves many functions. One function is to protect developing germ cells from toxicants and immune attacks [57]. Research in other mammals indicates that exposure to MP can degrade this barrier and disrupt its function [57]. In a recent study on human testes, researchers detected MP internalizing into Sertoli cells and disrupting the blood testes barrier, compromising sperm quality [10]. This study showed a significantly lower particle presence in the testes versus in the uterus plus ovaries, which could possibly be related to this barrier, even though microfibers have been documented to move through it [57]. Another possible cause for a greater presence of particles in uterine and ovarian tissue could be attributed to the overall greater amount of blood flow through the female reproductive system than through testicular tissue [58,59]. With feline blood known to carry MP [8], it is possible that the increased blood flow throughout the uterine tissue transports and deposits more particles than in the testicles.

Although some ingested plastics and anthropogenic particles are excreted in feces [60], particles also may become trapped in tissues or translocate through the bloodstream, resulting in the retention of particles in feline reproductive tissues [61]. The results of this study demonstrated that there was a significant interaction between age and tissue type for particle presence in domestic feline reproductive tissue. In testes, the probability of particle presence increased significantly with age; however, since particles were present in all domestic uteri and ovaries, no relationship between age and particle presence was able to be detected. It is still important to keep in mind that the domestic felines included in our study were all only in the range of 3–60 months old, with an average age of 13.8 ± 1.8 months. This restricted range of ages did not allow us the scope to understand how contaminant densities may fluctuate throughout the full lifespan of *F. catus*.

With this being a strictly observational study assessing the presence of particles in the reproductive tissue of felines, limitations arise as there was no experimentation done to explore the possibilities of these hypothetical exposure sources or possible implications. Further research will need to be done on this species to determine if sources such as diet, food storage containers, drinking water, aerial contamination, and toys could be contributors to MP entering reproductive tissue. Similarly, more testing will need to be done to see if any implications arise from plastics or AP being found in the reproductive tissues in male and female felines, as have been seen in other mammals.

While this study evaluated contaminants in the tissues of felines, this species is not alone in facing environmental and urban contaminants. One Health is an approach that recognizes the interconnection of humans, animals, and the environment [62]. In companion animals, contamination by metals, pesticides, and tobacco smoke has been examined [63,64,65,66]. By far, most studies have focused on plastic contamination, especially MP, as plastics have become so integrated into biological cycles and ecosystems worldwide [67]. These contaminants could have impacts on humans, animals, and plants present in those systems that have not yet been uncovered, emphasizing the importance of studying them and collaborating across disciplines to approach these matters. Humans live closely among felines, whether they are domestic house cats or feral cats living in neighborhoods. This could result in shared exposure to many of the same contaminants, emphasizing the importance of studying felines, as they experience both environments and can aid in studying health implications on humans and other mammals. In a study comparing MP concentrations in the testes of canines and the testes of humans, researchers saw MP in both species, with MP levels of 122.63 µg/g in dogs and 328.44 µg/g in humans [68]. They also noted that both humans and canines exhibit relatively similar proportions of the major polymer types [68], possibly pointing to similar exposure routes. This One Health approach can be utilized in further studies to uncover sources and implications these contaminants have on humans, animals, and ecosystems.

## 5. Conclusions

The results of this study indicated that 71% of 100 felines examined in this study had foreign particles present in their reproductive tissues. Feral felines were found to have significantly less MP present than domestic felines, and testes trended toward having less particle presence than the ovaries plus uteri. PET was the most prevalent polymer observed throughout and was identified in all four feline groups. It was also demonstrated that age did significantly influence particle presence in domestic feline reproductive tissue. In testes, the probability of particle presence increased significantly with age; however, since particles were present in all domestic uteri and ovaries, no relationship between age and particle presence was able to be detected. While more research is needed to uncover all the possible implications of MP contamination in feline reproductive tissue, the retention of these particles has resulted in harmful effects noted in both the uteri and ovaries as well as the testes of other mammals. Reproductive health is not only crucial for the health of felines now, but also for any resulting generations. Further studies will need to be done to look at all the implications MP have on feline tissue and develop strategies to address concerns. This study provides a baseline for MP concentrations in the reproductive tissue of both feral and domestic felines.

## Figures and Tables

**Table 1:** Feline Group Metrics. MP = microplastics. MSP = mesoplastics. AP = anthropogenic particles. Kg = kilograms. Pooled reproductive tissue weight = Females (uterus + ovaries), Males (both testes). FTIR = Fourier-Transform Infrared Spectroscopy. No library match = spectrum not detected in library.

Feline Group	Domestic Females	Domestic Males	Feral Females	Feral Males	Total
Number of Individuals	25	25	25	25	100
Age Range (months)	3–60	3–36	-	-	3–60
Feline Body Weight Averages ± SE (kg)	3.12 ± 0.15	3.77 ± 0.18	2.30 ± 0.10	3.54 ± 0.29	3.18 ± 0.19
Pooled Reproductive Tissue Weight Averages (grams)	3.62 ± 0.50	3.04 ± 0.31	3.60 ± 0.82	3.32 ± 0.38	3.39 ± 0.27
# Individuals Containing Particles (% of group)	25 (100%)	17 (68%)	20 (80%)	9 (36%)	71 (71%)
Particle Types (#)	Fiber: 97 Fragment: 1 Film: 1	Fiber: 35 Fragment: 2 Film: 0	Fiber: 68 Fragment: 1 Film: 1	Fiber: 14 Fragment: 0 Film: 0	Fiber: 214 Fragment: 4 Film: 2
Total FTIR-tested Particles (#)	25	21	33	12	91
FTIR-validated Particles	MP: 1 MSP: 0 AP: 10 Cellulose: 6 No Library Match: 8	MP: 5 MSP: 1 AP: 6 Cellulose: 6 No Library Match: 3	MP: 3 MSP: 7 AP: 22 Cellulose: 1 No Library Match: 0	MP: 1 MSP: 2 AP: 5 Cellulose: 4 No Library Match: 0	MP: 10 MSP: 10 AP: 43 Cellulose: 17 No Library Match: 11
Avg. FTIR- validated MP Length (mm) ± SE	2.00	1.50 ± 0.22	2.33 ± 0.67	1.50	1.80 ± 0.24
Avg. FTIR- validated MSP Length (mm) ± SE	-	6.00	8.00 ± 1.09	7.50 ± 1.50	7.70 ± 0.80
Avg. FTIR- validated AP Length (mm) ± SE	2.00 ± 0.32	2.02 ± 0.83	6.19 ± 1.08	5.20 ± 1.88	4.85 ± 0.65
Avg. FTIR- validated Cellulose Length (mm) ± SE	2.50 ± 0.34	2.42 ± 0.41	1.50	6.00 ± 1.41	3.10 ± 0.52

**Table 2:** Fourier-Transform Infrared Spectroscopy (FTIR) results of plastic and anthropogenic materials. MP = microplastics. MSP = mesoplastics. AP = anthropogenic particles.

Match Material: Plastic Polymers	n (% of MP)	n (% of MSP)
Polyethylene terephthalate (PET)	5 (50%)	4 (40%)
Polyurethane (PU)	3 (30%)	2 (20%)
Polyethylene	1 (10%)	1 (10%)
Polyester	-	2 (20%)
Poly (acrylic acid/starch) grafted resin	1 (10%)	1 (10%)
**Match Material: Anthropogenic Particles**	**n (%) of AP**	
Cellulose Derivative (Carboxymethyl cellulose)	20 (47%)	
Cellulose Derivative (Hydroxyethyl cellulose)	15 (35%)	
Thickener L (Poly methyl vinyl ether/maleic anhydride)	5 (11%)	
Rayon-absorbent	3 (7%)	
**Match Material: Cellulose**	**n (% of total Cellulose)**	
Cellulose	7 (41%)	
Kayocel 10W80	4 (24%)	
Alpha-cellulose	2 (12%)	
Cellulose (20 micron)	1 (6%)	
Cellulose (50 micron)	1 (6%)	
Regenerated cellulose	1 (6%)	
Kayocel LC 50	1 (6%)	

## Data Availability

Post-publication, all data will be available on the University of Central Florida STARS digital repository at https://stars.library.ucf.edu/hut2024/488/, accessed on 30 August 2026.

## Abbreviations

The following abbreviations are used in this manuscript:

MP	Microplastics
MSP	Mesoplastics
AP	Anthropogenic Particles
FTIR	Fourier-Transform Infrared Spectroscopy
µm	Micrometer
